# Leader ethical voice and subordinate job performance: the chain mediating role of subordinate identification with leader and leader-member exchange

**DOI:** 10.3389/fpsyg.2024.1340769

**Published:** 2024-06-19

**Authors:** Fubin Xia, Ping Lu, Lifang Wang

**Affiliations:** ^1^School of Economics and Management, Hanshan Normal University, Chaozhou, China; ^2^School of Education Science, Hanshan Normal University, Chaozhou, China; ^3^School of Business Administration, Dongbei University of Finance and Economy, Dalian, China

**Keywords:** leader ethical voice, subordinate identification with leader, leader-member exchange, task performance, social identity, social exchange

## Abstract

**Introduction:**

Ethical voice is a valuable ethical behavior that enables organizations to promptly recognize and rectify unethical issues and practices, thus preventing severe dilemmas and crises. Despite its importance, the extant literature has yet to fully explore the impact of a leader’s ethical voice on subordinate outcomes. This study bridges this gap by integrating social identity theory and social exchange theory to scrutinize the process by which a leader’s ethical voice affects subordinate task performance.

**Methods:**

We employ a serial mediation model to explore the mechanisms by which a leader’s ethical voice enhances subordinates’ task performance. Our theoretical framework is empirically validated using a dataset that includes 449 subordinate-leader pairings from Chinese enterprises.

**Results:**

The survey results demonstrate that a leader’s ethical voice has a significant positive impact on subordinate task performance. Subordinate identification with leader and leader-member exchange not only individually mediate the effects of a leader’s ethical voice on subordinate task behavior but also jointly serve as a chain-mediated mechanism in the influence of a leader’s ethical voice on subordinate task behavior.

**Discussion:**

These findings illuminate the substantial effects that ethical leadership behaviors exert on employee performance and offer fresh perspectives on the intricate dynamics that govern this influence.

## 1 Introduction

The widespread application of technologies such as the internet, Internet of Things (IoT), cloud computing, and big data has led to increasingly rapid changes in the organizational environment. This poses new challenges to organizational ethical practices, requiring organizations to adapt their ethical standards, policies, and other aspects to meet the demands of ethical realities. In recent years, there have been numerous cases of business ethics scandals, which are closely related to this issue. For example, the 2020 fraud scandal of Chinese company Luckin Coffee, the 2020 bankruptcy of German Alipay company Wirecard, and the bankruptcy of cryptocurrency exchange FTX in March 2023, among others. These events illustrate that unethical practices not only cause significant losses to the companies involved but also pose serious harm to society as a whole. Moreover, a nationwide survey conducted by Yale University on over 14,500 employees in various industries in the United States also revealed the widespread presence of unethical practices in businesses, with approximately one-fourth of the surveyed employees feeling pressured to do things they know are wrong (Ivcevic et al., 2020). In this context, it is imperative for organizational members, particularly leaders, to challenge and seek to change existing unethical behaviors, procedures, and policies within their organizations in order to enhance the overall ethical climate.

In recent years, scholars have been at the forefront of exploring the ethical behaviors undertaken by organizational members to enhance ethical standards within organizations. One concept that has emerged is ethical voice, which refers to the open discussion and opposition of unethical issues and phenomena within an organization by its members (Huang and Paterson, 2017; Lee et al., 2017). The purpose of Ethical Voice is to challenge and change the organization’s unethical behaviors, procedures, and policies (Huang and Paterson, 2017). It is a valuable ethical behavior that helps organizations timely identify and address existing unethical problems and practices, thereby avoiding serious issues and crises (Lee et al., 2017; Zheng et al., 2021).

Ethical voice is not limited to subordinates, and leaders can also voice their concerns to address ethical issues within the organization (Paterson and Huang, 2019; Faheem et al., 2021). Due to their roles and positions, leaders often have a greater influence in promoting ethical practices compared to subordinates (Epitropaki et al., 2017). More importantly, the moral influence demonstrated by the leader’s behavior can not only inspire subordinates to exhibit a higher level of morality (Epitropaki et al., 2017), but it may also further affect their job performance. However, existing research on ethical voice has mainly examined how different leadership styles, such as moral leadership (Huang and Paterson, 2017; Lee et al., 2017; Afsar and Shahjehan, 2018; Kim and Vandenberghe, 2020; Alshehri and Elsaied, 2022; Zheng et al., 2022), authentic leadership (Froemmer et al., 2021), autocratic leadership (Zheng et al., 2021), social responsibility human resource management (Zhao et al., 2023), and individual moral transgression (Xia et al., 2023), affect employees’ ethical voice. Little research has been conducted on the influence of leader’s ethical voice on subordinates job behaviors, which limits our understanding of the positive effects of leader’s ethical voice and its significant value within organizations.

This research is anchored in social exchange theory (Tajfel and Turner, 2004) and social identity theory (Blau, 1964), which are pivotal for understanding leadership effectiveness, as demonstrated in leadership studies (Epitropaki et al., 2017; Peng and Kim, 2020; Hou et al., 2023). Although extensive research has shown that leaders can shape subordinates’ work-related attitudes and behaviors through leadership identification and leader-member exchange dynamics (Götz et al., 2020; Vriend et al., 2020; Banks et al., 2021), the integration of social identity theory with social exchange theory to probe leaders’ influence on subordinates’ psychological processes remains underexplored. Moreover, the literature lacks an examination of how leaders’ ethical voice affects subordinates’ performance within these theoretical frameworks. This study aims to fill this gap by proposing various mechanisms by which a leader’s ethical voice can enhance subordinates’ job performance, drawing on insights from social identity theory (Tajfel and Turner, 2004) and social exchange theory (Blau, 1964).

Firstly, based on social identity theory, leaders can influence subordinates’ attitudes and behaviors by shaping their self-identification (e.g., Epitropaki et al., 2017; Peng and Kim, 2020). Therefore, this study constructs a mediation model “leader’s ethical voice - subordinates identification with leader - task performance” to explain the social identity mechanism by which a leader’s ethical voice affects subordinates’ job performance.

Secondly, according to social exchange theory, leaders can implement effective leadership by improving the leader-member exchange relationship (e.g., Epitropaki et al., 2017; Banks et al., 2021). Therefore, this study constructs a mediation model “leader’s ethical voice - leader-member exchange - task performance” to explain the social exchange mechanism through which leader’s ethical voice influences subordinates job performance.

Finally, this study proposes that subordinates identification with leader and leader-member exchange might mutually influence one another when guided by leader’s ethical voice. It is hypothesized that subordinates, out of admiration and recognition for leader’s ethical voice, develop identification towards their leaders, which in turn enhances the development of high-quality exchange relationships with their superiors. This hypothesis is based on the logic that leader-member exchange is a two-way relationship based on behavioral interactions (Bauer and Green, 1996), whose quality depends on mutual trust, recognition, and respect (Graen and Scandura, 1987). On the other hand, subordinates’ leader identification differs in nature, as it is a one-way identification based on leaders’ values and behaviors (Pratt, 1998), reflecting subordinates’ recognition, trust, and loyalty towards their leaders (Shamir et al., 1998; van Quaquebeke and Eckloff, 2010). This implies that subordinates may directly develop identification with their superiors due to their admiration and recognition of leader’s ethical voice, which provides the cues necessary to establish high-quality relationships. Therefore, this study proposes a mediated chain model “leader’s ethical voice - subordinates identification with leader - leader-member exchange - task performance” to further explain how leader’s ethical voice influences subordinates job performance through the combined mechanisms of social identification and exchange.

This study makes two contributions. Firstly, it examines the relationship between leader’s ethical voice and subordinates task behaviors, furthering the understanding of the effects of leader’s ethical voice and enriching the field of ethical voice research. Secondly, it reveals the multiple underlying mechanisms through which leader’s ethical voice influences subordinates job performance, elucidating the internal logic behind the positive effects of leader’s ethical voice and contributing to the understanding of how leaders can influence organizational efficiency through their ethical behavior. These contributions are of great significance in comprehensively and deeply understanding the effectiveness of leader’s ethical voice, innovating leadership ethics theories, and promoting organizational ethical practices.

We begin by formulating research hypotheses grounded in social identity theory and social exchange theory. Subsequently, we elaborate on our research methodology and present our findings. Ultimately, we undertake a comprehensive analysis of the results, culminating in the articulation of our research conclusions.

## 2 Theory and hypothesis development

### 2.1 Leader’s ethical voice and subordinates task performance

The moral behavior of leaders is a core factor in many leadership theories explaining leadership effectiveness and is seen as key to leaders exerting influence and guiding subordinates’ attitudes and behaviors (e.g., Brown et al., 2005; Fehr et al., 2015). Leader’s ethical voice is a behavior that openly challenges and seeks to change the moral status quo of the organization, with clear moral connotations and high risk (Paterson and Huang, 2019). Therefore, as a special type of leader’s moral behavior, leader’s ethical voice may have a greater impact on subordinates’ work behavior.

This judgment is made because, according to the moral foundation theory (Fehr et al., 2015), compared to other types of leaders’ moral behavior (such as caring for subordinates), leader’s ethical voice better reflects the leaders’ moral quality and sense of responsibility. For example, when leaders speak out about immoral phenomena and problems within the organization, they may face opposition, dissatisfaction, and retaliation from others, causing interpersonal conflicts, and even jeopardizing their own positions (Lee et al., 2017). Therefore, when leaders show this kind of courage and behavior of “advising at the risk of death”, subordinates may more recognize their moral quality and sense of responsibility (Fehr et al., 2015). Consequently, it can be inferred that among many types of leaders’ moral behavior, the sincere concern and responsibility for the organization and subordinates demonstrated by leaders through ethical voice can more effectively stimulate subordinates’ positive responses.

Subordinates’ positive responses may be manifested in that they will take more initiative to make work behaviors beneficial to the organization and leaders, in order to reward and support the leader’s integrity. Task behavior refers to those behaviors that directly serve organizational goals and produce the desired results (Motowidlo and Van Scotter, 1994), and are the basic job responsibilities of employees (Koopmans et al., 2011). Task behavior is an important driver of organizational performance and is a necessary and key employee work behavior for the survival and development of the organization. Therefore, under the inspiration of the leader’s ethical voice, employees will strive to complete their own work (task behavior).

### 2.2 The mediating role of subordinates identification with leader

Self or identity mainly includes personal self (identity), relational self (identity), and group self (identity) (Sedikides et al., 2011), which will affect individuals’ emotions, attitudes, and behaviors (Owens et al., 2010). Subordinates identification with leader is a kind of relational self-identification, reflecting to what extent subordinates define themselves by the characteristics of leaders (Ashforth et al., 2016). When employees find that they have common core values and beliefs with leaders, or when subordinates are willing to adjust their values and beliefs to be closer to leaders, they often identify with leaders (Pratt, 1998).

Based on social identity theory (Tajfel and Turner, 2004), the ethical voice of leaders can increase subordinates identification with the leader. To elaborate, we argue that leaders who persist in ethically voicing for the benefit of the organization and employees despite facing risks and personal losses are perceived by subordinates as a person with responsibility, sincerity, and altruism. This perception not only boosts the subordinates’ respect and endorsement for the leader’s moral behavior and underlying values, but also strengthens the subordinates’ trust and appreciation for the leader’s sincere and upright character. Even when a leader’s ethical voice may not be fully comprehended by their subordinates, they still perceive the leader as a person of trustworthiness, reliability, and fairness, who consistently adheres to moral standards in all endeavors (Lee et al., 2017). These two types of “value-oriented” and “character-oriented” moral cognition can motivate subordinates to admire, align with, and accept their superiors, thereby encouraging them to approach their superiors more closely and aspire to become a person similar to their superiors in terms of values and behavior (Pratt, 1998). Therefore, when expressing moral positions in a self-sacrificing manner, leaders can effectively narrow the psychological distance with subordinates and improve their psychological identification with themselves. Based on the above analysis, this study proposes the following hypothesis:

H1: Leader’s ethical voice positively affects subordinates identification with leader.

Previous studies have confirmed the positive impact of subordinates identification with leader on their work behavior (e.g., Walumbwa and Hartnell, 2011; Gu et al., 2015). Therefore, this study hypothesized that subordinates identification with the leader enhanced through leader’s ethical voice would lead to more proactive behavior in their work. On one hand, after building leader identification, subordinates consider the leader’s interests as mutual interests, trust and lean on them, and are willing to put more effort in their work to display support for the leader’s ethical voice (Van Knippenberg et al., 2005). On the other hand, in the subordinates’ perspective, leaders usually represent the organization. Therefore, subordinates identification with their supervisor implies their willingness to embrace and follow organizational goals, behavioral norms, and values (Walumbwa and Hartnell, 2011), and perform task behaviors that match organizational expectations. Consequently, high levels of leader identification resulting from ethical voice may promote task behavior in subordinates. These two aspects indicate that subordinates’ identification with their supervisors, driven by the leader’s ethical voice, may simultaneously promote their task behavior. In conjunction with H2, this study proposes the following hypothesis:

H2: Subordinates identification with leader mediates the relationship between leader’s ethical voice and subordinate task performance.

### 2.3 The mediating role of leader-member exchange

Leader-member exchange refers to the social exchange relationship between leaders and subordinates based on mutual trust and reciprocity (Graen and Uhl-Bien, 1995). The quality of the relationship reflects different social exchange patterns between the two parties. Low-level leader-member exchange involves economic exchanges defined by formal employment contracts, while high-level leader-member exchange goes beyond economic exchanges to include the exchange of social and emotional resources (Liden et al., 1997). The quality of leader-member exchange depends on the extent of mutual trust, respect, and appreciation between leaders and subordinates (Graen and Scandura, 1987; Gu et al., 2015).

Based on social exchange theory (Blau, 1964), a leader’s ethical voice can enhance the quality of leader-member exchange. The main reason is that a leader’s ethical voice can foster trust, respect, and appreciation between leaders and subordinates, thereby enhancing the quality of their social exchange relationship. Specifically, when supervisors voice their ethical concerns, they not only demonstrate a noble spirit of “sacrificing oneself for the greater good”, but also show loyalty and a sense of responsibility to the organization and employees, as well as sensitivity and courage in addressing moral or ethical issues. Such supervisors have a high level of moral character, are honest, respectful, caring, and treat their subordinates fairly (Bedi et al., 2016). Therefore, subordinates trust and respect such supervisors. Moreover, subordinates’ trust, reliance, and respect for their supervisors also motivate them to actively behave in ways that meet the expectations of the leader and the organization, thereby supporting and rewarding the supervisor (Martin et al., 2016), further enhancing the trust and respect that the supervisor has for them. In this way, the emotional bond and economic benefits between the two parties in the social exchange process can be effectively maintained and strengthened. Based on this, the following hypothesis is proposed in this study:

H3: Leader’s ethical voice positively influences leader-member exchange.

After strengthening the relationship quality between subordinates and leaders through leader’s ethical voice, subordinates may repay and support the leader by improving task performance. There are two main reasons for this. Firstly, high-quality leader-member exchange implies a sense of obligation and reciprocity between both parties (Dulebohn et al., 2012). Therefore, subordinates feel obligated and responsible to work harder and go the extra mile to repay and support the leader after their ethical voice, by improving task performance. Secondly, leader-member exchange itself is a motivating factor for employees (Tierney et al., 1999). Subordinates’ positive emotions such as trust, dependence, and respect for the leader resulting from leader’s ethical voice can motivate them to focus on meeting the leader’s expectations and needs, thereby improving their task behaviors within their roles. This analysis shows that leader-member exchange can have a positive impact on subordinates’ task behaviors. In alignment with H3, this study puts forth the subsequent hypothesis:

H4: Leader-member exchange mediates the relationship between leader’s ethical voice and subordinate task performance.

### 2.4 The chain mediating role of subordinates identification with leader and leader-lember exchange

The core of leader-member exchange lies in the reciprocal exchange of resources between the two parties (Sparrowe and Liden, 1997), and subordinates identification with leader strengthens this reciprocity due to leader’s ethical voice. There are two reasons for this: first, subordinate leader identification leads them to believe that the social exchange with the leader is a win-win process, where sharing resources can create greater value (Creary et al., 2015). Second, subordinate identification with the leader implies that they perceive the leader as trustworthy, honest, and selfless (Edmondson, 1999), which eliminates concerns about exploitation and unequal exchange, thereby enhancing psychological safety (Nembhard and Edmondson, 2006). Thus, subordinates identification with leader contributes to the establishment of positive, equal, safe, and reciprocal resource exchange relationships, which positively influence leader-member exchange. Based on the above hypotheses (H1,H2,H3,H4), this study posits that leader’s ethical voice can improve the relationship quality between leaders and subordinates by enhancing subordinate identification with leader, thereby facilitating subordinate task behavior. Therefore, this study further proposes the following hypothesis:

H5: Subordinate identification with leader and leader-member exchange play a chain mediating role in the relationship between leader’s ethical voice and subordinate task performance.

**FIGURE 1:**
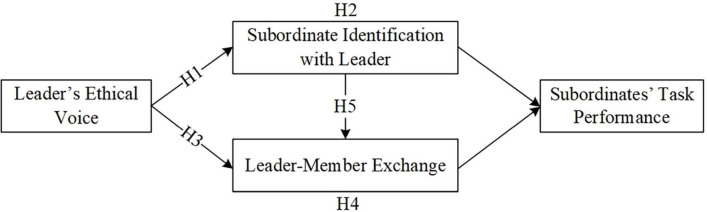
Hypothesized research model.

Based on the above theoretical analysis, we propose the research model shown in Figure 1.

## 3 Materials and methods

### 3.1 Participants and procedures

This study used a paired questionnaire survey method to collect data from twenty-five enterprises across diverse industries in China in July and August 2023. The sectors surveyed encompassed manufacturing, construction, the financial industry, as well as an array of service-oriented sectors, among others. The questionnaires were sent by email to the respondents. After obtaining the consent of the leaders of the target companies, the Human Resources department collected voluntary participation from employees and their direct supervisors in a one-to-one matched manner, and collected their email information. After organizing the paired email directory, we sequentially sent the corresponding supervisor and subordinate questionnaires to each paired email. To ensure data matching, both the supervisor and subordinate questionnaires were labeled with the same three-digit identification code. Among them, the subordinate’s background information, leader’s ethical voice, subordinate identification with leader, and leader-member exchange were evaluated by the subordinates, while subordinate task performance was evaluated by their supervisors.

A total of 580 leader-subordinate paired questionnaires were sent out. Following the removal of questionnaires that were incomplete, incongruent, or filled out in a seemingly random manner, we successfully compiled 449 valid paired responses, achieving an effective response rate of 77.41%. Among them, males accounted for 45.4% and females accounted for 54.6%. In terms of age, 27.6% were 25 years old or younger, 47.0% were between 26 and 35 years old, 20.3% were between 36 and 45 years old, and 5.1% were 46 years old or older. In terms of education level, 23.6% had high school education or below, 21.9% had a college degree, 45.0% had a bachelor’s degree, and 13.6% had a master’s degree or higher. In addition, in terms of work experience, 21.8% had less than 1 year, 27.2% had 1–3 years, 19.2% had 4–6 years, and 31.9% had more than 6 years of work experience.

### 3.2 Measures

This study adopted established and widely used scales as measurement tools. The English scales were translated and back-translated following a standard procedure to ensure their reliability and validity in the Chinese context (Brislin, 1986). The scales were also verified by professionals in this study. All scales used a Likert 5-point rating scale from 1 (completely disagree) to 5 (completely agree).

#### 3.2.1 Leader’s ethical voice

We used the leader’s ethical voice scale developed by Zheng et al. (2022), and modified some terms to fit our research needs. A sample item is “My supervisor speaks up in the organization to stop others from behaving with a lack of integrity”. The Cronbach’s alpha of this scale in our study was 0.865.

#### 3.2.2 Subordinate identification with leader

The organizational identification scale developed by Mael and Ashforth (1992) was used, with the measurement target changed from “organization” to “leader”. It included items such as “My supervisor’ success is my success”. The Cronbach’s alpha for this study was 0.876.

#### 3.2.3 Leader-member exchange

The leader-member exchange scale adapted from Zhao et al. (2014) based on Wang et al. (2005) was used. One of item of leader-member exchange scale is “I maintain a positive and effective working relationship with my supervisor”. The Cronbach’s alpha for this study was 0.894.

#### 3.2.4 Task performance

The task performance scale developed by Dyne and LePine (1998) was used, including items such as “This particular employee adequately completes responsibilities”. The Cronbach’s alpha for this study was 0.920.

#### 3.2.5 Control variables

To effectively control the impact of age, gender, education level, and work experience on the research results, this study treated them as control variables. It has been substantiated that these control variables can potentially exert an influence on the work outcomes of employees (Ng and Feldman, 2008).

## 4 Results

### 4.1 Descriptive statistics

Table 1 presents the means, standard deviations, and correlation coefficients of the variables in this study. The correlation analysis results indicate significant correlations among the research variables, providing preliminary support for our theoretical expectations.

**TABLE 1:** Means, standard deviations, and correlation coefficients of the variables.

Variables	M	SD	1	2	3	4	5	6	7	8
1 Gender	1.550	0.498	1							
2 Age	2.030	0.827	−0.304**	1						
3 Education Level	2.540	0.937	0.296**	−0.245**	1					
4 Work Experience	2.840	1.455	−0.399**	0.533**	−0.294**	1				
5 LEV	3.371	1.072	0.070	−0.091	0.002	−0.046	1			
6 SIL	2.895	0.929	−0.030	0.050	−0.056	0.084	0.415**	1		
7 LMX	3.172	0.854	0.040	0.001	−0.007	0.022	0.592**	0.667**	1	
8 TP	3.909	0.900	0.082	−0.027	0.056	−0.023	0.210**	0.194**	0.325**	1

N = 449;* *p <* 0.05 ** *p* < 0.01*** *p* < 0.001; LEV = Leader’s ethical voice; SIL, Subordinate identification with leader; LMX, Leader-member exchange; TP, Task performance.

### 4.2 Measurement model

We tested common method bias using Harman’s one-factor test and latent method factor. First, The Harman’s test showed that the single factor explained 37.873% (< 40%) of the total variance. Second, following the recommendations of Podsakoff et al. (2003), the method of controlling for unmeasured latent method factors was used to further test for common method bias. The results showed that after incorporating the common method bias as a latent variable in the CFA model to construct a common method factor model, there was no significant improvement in the fit indices of the model (△CFI = 0.021, △TLI = 0.018, △RMSEA = 0.009, △SRMR = 0.014). Thus, common method bias was not a major issue for the data collection in this study.

Discriminant validity was assessed using Fornell and Larcker’s method. According to the Fornell-Larcker criterion (Fornell and Larcker, 1981), the square root of the Average Variance Extracted (AVE) for each construct must surpass the respective inter-construct correlation values. As depicted in Table 2, this condition is met, indicating the absence of reliability concerns.

**TABLE 2:** Model of discriminant validity.

	LEV	SIL	LMX	TP
LEV	0.789			
SIL	0.413***	0.712		
LMX	0.565***	0.574***	0.707	
TP	0.189***	0.142***	0.258***	0.877

N = 449; * *p <* 0.05 ** *p* < 0.01*** *p* < 0.001; Numbers on the diagonal show square roots of AVE. Numbers below the diagonal show the factor correlations; LEV, Leader’s ethical voice; SIL, Subordinate identification with leader; LMX, Leader-member exchange; TP, Task performance.

Confirmatory factor analyses were performed to evaluate the construct discrimination of the research variables. The fit indices presented in Table 3 reveal that the four-factor model outperformed the alternative models. This suggests robust discriminant validity among the four research variables. Additionally, the findings imply that the measurement of these constructs is not substantially affected by common method bias.”

**TABLE 3:** Results of confirmatory factor analysis for the structural validity of variables.

Models	χ2	df	χ2/df	RMSEA	CFI	TLI	SRMR
LEV SIL LMX TP	521.169	183	2.848	0.064	0.938	0.929	0.047
LEV SIL+LMX TP	795.896	186	4.279	0.085	0.888	0.873	0.063
LEV+LMX TP SIL	917.756	186	4.934	0.094	0.865	0.848	0.066
LEV+SIL LMX TP	1118.306	186	6.012	0.106	0.828	0.806	0.076
LEV LMX SIL+TP	1921.845	185	10.388	0.144	0.68	0.639	0.117
LEV SIL LMX+TP	1803.604	186	9.697	0.139	0.702	0.664	0.110
LEV+SIL+TP LMX	2477.836	188	13.180	0.165	0.579	0.529	0.128
LEV+LMX +TP SIL	2408.933	188	12.813	0.162	0.591	0.546	0.155
LEV+LMX SIL +TP	1435.306	188	7.635	0.121	0.771	0.745	0.127
LEV+SIL+LMX TP	1256.146	188	6.682	0.112	0.803	0.780	0.079
LEV+SIL+LMX+TP	2581.152	189	13.657	0.168	0.56	0.511	0.129

“+” Indicates factors combined; LEV, Leader’s ethical voice; SIL, Subordinate identification with leader; LMX Leader-member exchange; TP, Task performance.

### 4.3 Hypothesis tests

First, After controlling for gender, age, education level, and work experience, the results of the linear regression analysis show that leader’s ethical voice has a significant positive predictive effect on subordinate task performance (β = 0.174, p < 0.001).

Second, Table 4 provides the bootstrap analysis results of the independent mediating effects of subordinate identification with leader and leader-member exchange (Bolin, 2014). The results revealed that the leader’s ethical voice positively influenced subordinate identification with leader (β = 0.368, p < 0.001) and leader-member exchange (β = 0.475, p < 0.001), confirming Hypotheses 1 and 3, respectively. Subordinate identification with leader (β = 0.130, p < 0.01) and leader-member exchange (β = 0.325, p < 0.001) positively predicted task performance. Moreover, the indirect effect of the leader’s ethical voice on task performance through subordinate identification with leader was 0.048, with a 95% confidence interval of [0.008, 0.094], supporting Hypothesis 2. Similarly, the indirect effect of the leader’s ethical voice on task performance through leader-member exchange was 0.155, with a 95% confidence interval of [0.090, 0.220], confirming Hypothesis 4.

**TABLE 4:** Independent mediation effects bootstrapping results.

Paths and effects	SIL mediation effect				LMX mediation effect			
	Effect	BootSE	Boot 95% CI		Effect	SE	Boot 95% CI	
Total Effect	0.174	0.047	0.083	0.266	0.174	0.047	0.084	0.267
Direct Effect	0.126	0.055	0.018	0.233	0.019	0.055	−0.090	0.129
Indirect Effect	0.048	0.022	0.008	0.094	0.155	0.033	0.090	0.220

SIL, Subordinate identification with leader; LMX, Leader-member exchange.

Finally, this study used bootstrapping to analyze the multiple mediation effects of subordinate identification with leader and leader-member exchange (Bolin, 2014). The results presented in Table 5 demonstrate that the overall impact of ethical leadership voice on subordinate task performance is statistically significant (0.174), with a 95% confidence interval of [0.083, 0.265]. The total indirect effect is also significant (0.153), with a 95% confidence interval of [0.088, 0.222]. Moreover, the chained multiple mediation effect of leader’s ethical voice on task performance through subordinate identification with leader and leader-member exchange is significant (0.060), with a 95% confidence interval of [0.031, 0.095], confirming Hypothesis 5.

**TABLE 5:** Multiple mediation effects bootstrapping results.

Paths and effects	Effect	BootSE	Boot 95% CI	
Total Effect: LEV → TP	0.174	0.047	0.083	0.265
Direct Effect: LEV → TP	0.020	0.057	−0.091	0.133
Total Indirect Effect:	0.153	0.034	0.088	0.222
Path 1: LEV → SIL → TP	−0.012	0.024	−0.059	0.035
Path 2: LEV → LMX → TP	0.106	0.026	0.057	0.156
Path 3: LEV → SIL → LMX → TP	0.060	0.016	0.031	0.095

LEV, Leader’s ethical voice; SIL, Subordinate identification with leader; LMX, Leader-member exchange; TP, Task performance.

## 5 Discussion

The ethical practices of organizational members have become an important pathway for business growth and gaining competitive advantages. This study integrates social identity theory and social exchange theory to examine the impact mechanism of high-risk, high-value ethical behavior, namely leader’s ethical voice, on subordinates task performance. Many studies have confirmed the influence of leadership behavior on subordinates’ work attitude and behavior (e.g., Lemoine et al., 2019). Positive leadership behavior can stimulate subordinates’ work enthusiasm and efficiency (e.g., McQuade et al., 2021; Zhang et al., 2022), while negative leadership behavior can weaken subordinates’ work motivation and performance (e.g., Fischer et al., 2021; Hassan et al., 2023). Our research corroborates these findings, demonstrating that a leader’s ethical voice significantly enhances subordinates’ task performance. This effect is mediated through two distinct pathways: subordinate identification with the leader and the quality of leader-member exchange. Diverging from prior studies, our research uncovers a chain mediation effect—a leader’s ethical voice boosts task performance by first strengthening subordinate identification with the leader, which in turn enriches leader-member exchange. Previous investigations have largely explored the influence of leaders on subordinates by separately applying social identity and social exchange theories (e.g., Peng and Kim, 2020; Banks et al., 2021; Hou et al., 2023). Our study, however, probes the complex interplay within these dynamics, offering a more intricate understanding of the leader’s impact on subordinate behavior. Overall, these findings confirm our research hypotheses and have positive implications for theoretical research and managerial practices.

### 5.1 Theoretical contributions

First, this study advances the understanding of the utility of leadership ethical behavior by exploring the impact of the leader’s ethical voice on the subordinates’ task performance. Currently, there is scarce research on the leader’s ethical voice, and its effectiveness remains ambiguous. By investigating the relationship between the leader’s ethical voice and the subordinates’ task performance, this study found that “leader’s ethical voice can effectively improve the subordinates’ task performance,” enriching the theoretical research on leadership ethical behavior and providing new insights into the value of leadership ethics.

Second, this study not only reveals the positive influence of leadership ethical voice on subordinates’ task performance but also uncovers its underlying mechanisms. Building upon the positive impact of leader’s ethical voice on subordinates’ task performance, this study integrates social identity theory and social exchange theory to explore the mechanisms of leader’s ethical voice on subordinates’ task performance. It not only discovers the social identity mechanism and the social exchange mechanism of leader’s ethical voice on subordinates’ task performance, but also further identifies the joint mechanism of social identity and social exchange. These new research findings enhance our understanding of the underlying mechanisms by which leader’s ethical behavior affects subordinates’ job performance, enrich the theoretical content of leadership ethical behavior, and provide theoretical support for future research.

### 5.2 Implications for practice

Leader’s ethical voice not only benefits the promotion of organizational ethical practices but also enhances subordinates’ effectiveness. Therefore, this study provides insights for organizations to effectively utilize leadership ethical behavior.

First, organizations can foster a conducive environment for the generation of leader’s ethical voice by providing comprehensive support, incentives, and protection mechanisms through policies, systems, and organizational resources (Zheng and Mao, 2021). An effective initiative is the ethical voice awards program, which serves not only to safeguard leaders but also to inspire them to confidently contribute their insights. Additionally, organizations can emphasize the value of leader’s ethical voice and help employees develop positive leader identification and leader-member relationships. By cultivating a culture that values ethics and practices within the organization, such as by promoting and rewarding leader’s ethical voice, employees can appreciate the importance of leadership ethical behavior and enhance their leader identification and leader-member exchange, which in turn improves their job performance. Moreover, it is imperative for leaders to engage in clear and impactful communication with their team members, steering them towards understanding and embodying the leaders’ ethical principles. Finally, cultivating the perception of leaders as moral exemplars is beneficial within an organization. This approach not only furnishes employees with a benchmark for ethical conduct but also motivates them to emulate such behavior.

### 5.3 Limitations and future research

Firstly, although this study used a multi-source data collection method by collecting self-report data from both supervisors and subordinates, which alleviated common method bias to some extent, future research could adopt methods such as multiple time points or experience sampling to enhance the rigor and explanatory power of the research. Secondly, the external validity of the research findings is limited, and future studies should expand the sample collection scope to further test the generalizability of the research findings. Thirdly, this study only explores the promotion mechanism of leader’s ethical voice on subordinates’ task behavior, and it is also necessary to investigate its impact on other work behaviors of subordinates. For example, considering the high-risk nature of leader’s ethical voice, future research could explore the relationship between leader’s ethical voice and subordinates’ prosocial deviant behavior, which also carries risks, and uncover the boundary conditions and underlying mechanisms of how leader’s ethical voice influences subordinates’ prosocial deviant behavior. Fourthly, this study did not explore the moderating effects of situational factors and individual differences on the role of leader’s ethical voice on subordinates’ task performance. Future research could consider exploring the moderating effects of factors such as corporate social responsibility and subordinates’ moral levels on the relationship between leader’s ethical voice and subordinates’ task performance. Finally, This study has definitively established that leaders’ ethical voice significantly influences subordinates’ task performance at the first-order factor level, mediated sequentially by subordinates’ identification with the leader and leader-member exchange. However, research conducted by Manata and Grubb (2022) has showed that the existing leader-member exchange measurement is second-order unidimensional. Our second-order factor analysis of the survey data provides novel evidence supporting this research conclusion, indicating that the second-order factor, comprising subordinate identification with the leader and leader-member exchange, mediates the relationship between a leader’s ethical voice and a subordinate’s task performance (The indirect effect is 0.140, with a 95% bootstrap interval between 0.058 and 0.235). Therefore, future investigations into the nexus between subordinates’ identification with the leader and leader-member exchange, especially from the vantage point of the second-order construct, hold substantial promise for advancing our understanding in this domain.

## 6 Conclusion

This study conducted an empirical analysis to examine the impact of leader’s ethical voice on subordinate task performance. The results revealed a significant positive influence of leader’s ethical voice on subordinate task performance. Furthermore, it was found that subordinate identification with the leader and leader-member exchange not only mediated the relationship individually but also jointly served as a chain of mediating factors. These findings shed light on the mechanisms through which leader’s ethical voice affects subordinate task performance. Consequently, our study contributes new insights into the effectiveness of leader’s moral behaviors.

## Data availability statement

The original contributions presented in the study are included in the article/Supplementary material, further inquiries can be directed to the corresponding author.

## Ethics statement

The studies involving humans were approved by the Hanshan Normal University ethics committee. The studies were conducted in accordance with the local legislation and institutional requirements. The participants provided their written informed consent to participate in this study.

## Author contributions

FX: Conceptualization, Data curation, Formal analysis, Funding acquisition, Investigation, Methodology, Project administration, Resources, Software, Supervision, Validation, Visualization, Writing–original draft, Writing–review and editing. PL: Conceptualization, Data curation, Formal analysis, Funding acquisition, Investigation, Methodology, Project administration, Resources, Software, Supervision, Validation, Visualization, Writing–original draft, Writing–review and editing. LW: Conceptualization, Data curation, Formal analysis, Investigation, Resources, Software, Supervision, Validation, Visualization, Writing–original draft, Writing–review and editing.
